# Successful removal of an intra-pericardic Kirschner wire via fluoroscopy-guided minimal invasive approach: a case report

**DOI:** 10.1186/s40792-023-01803-8

**Published:** 2024-03-11

**Authors:** Olivier Georges, Florence De Dominicis, Malek Ben Rahal, Jules Le Pessot, Pascal Berna, Osama Abou Arab, Christophe Beyls

**Affiliations:** 1 Department of Thoracic Surgery, Anesthesiology and Critical Care Medicine, Amiens University Hospital, 1, Rond Point du Pr Cabrol, 80054 Amiens Cedex 1, France; 2 Department of Thoracic Surgery, Clinique Victor Pauchet, Amiens, France; 3 Department of Anesthesiology and Critical Care Medicine, Amiens University Hospital, 80054 Amiens, France

**Keywords:** Kirchner wire, Pericardium, Thoracic surgery, Minimally invasive

## Abstract

**Background:**

Kirschner wires are widely used in trauma surgery. Their migration into the pericardium is a rare but often fatal phenomenon, requiring urgent management.

**Case presentation:**

We describe the case of a 65-year-old patient who underwent Kirschner wire placement to treat a humeral head fracture. Three months after the operation, pleural and pericardial effusions with cardiac tamponade were observed, leading to the diagnosis of wire migration within the pericardium. A minimally invasive approach guided by fluoroscopy allowed emergency wire extraction without needing a median sternotomy. The postoperative clinical course was uncomplicated.

**Conclusions:**

The use of pre- and per-operative multimodal imaging allowed for the safe extraction of an intra-pericardial Kirschner wire through a minimally invasive approach.

**Supplementary Information:**

The online version contains supplementary material available at 10.1186/s40792-023-01803-8.

## Introduction

The use of orthopedic metal pins, known as Kirschner wires (K-wires), is a standard surgical treatment of humerus and clavicle fractures. Despite an initial intraosseous positioning, pin migration is a well-known complication, with many case reports already published. However, most of these pins migrations remain localized to the peri-fracture zone and do not constitute a life-threatening situation for the patient [1]. Rarely, due to their shape and size, the pins can migrate much further and cause severe lesions, especially in case of migration to the thoracic cavity or even to the pericardium.

### Ethics

For the case we describe, we obtained study approval and informed written consent for publication from the patient, following the strengthening of reporting guidelines for case report studies CARE.

## Case

**Fig. 1:**
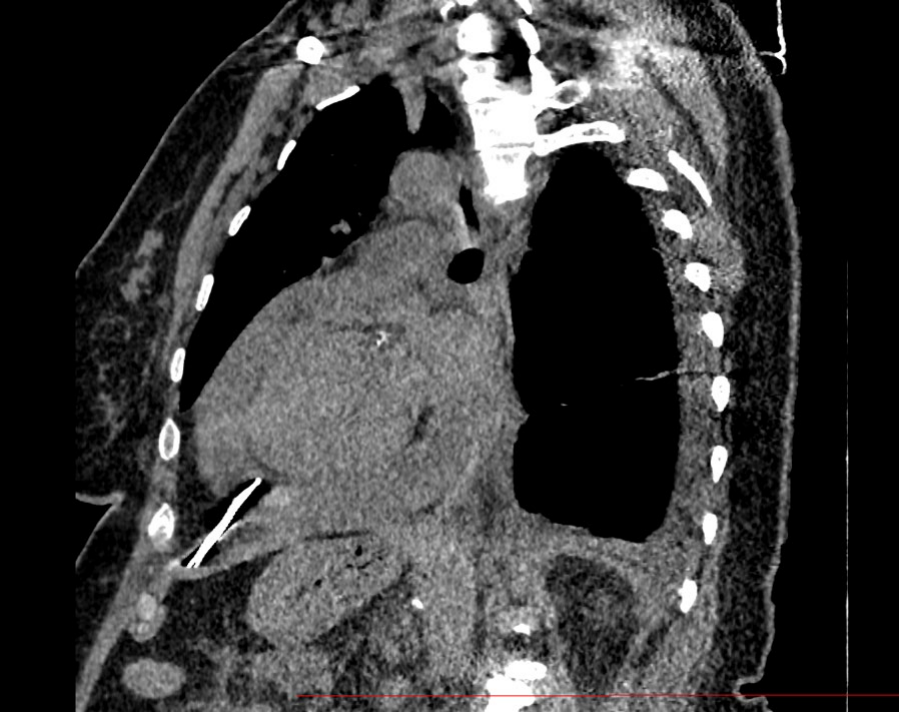
Sagittal view of a preoperative thoracic CT scan centered on the Kirschner wire without contrast

**Fig. 2:**
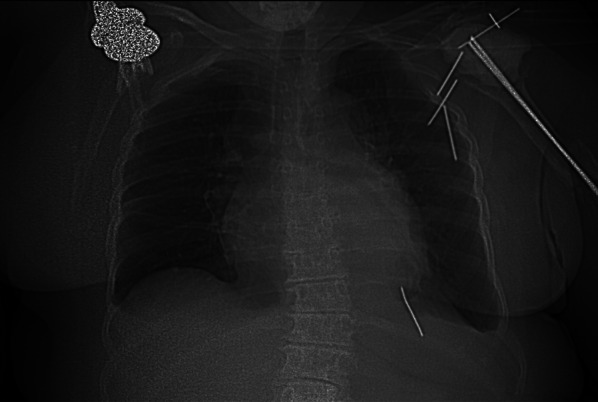
Scout view of preoperative thoracic CT scan

A 65-year-old female patient with a history of diabetes and severe obesity (BMI 42.1) underwent open reduction and internal fixation of a proximal humerus fracture using five straight K-wires. In the following, a 1-month repeat X-ray showed 3 K-wires had started to migrate toward the thoracic cavity. In the third month, the patient presented to the emergency department with exertional dyspnea of progressive onset. Clinical examination revealed mild lower limb edema without other signs of right or left heart failure, except for dyspnea. The ECG showed atrial fibrillation with a rapid ventricular response at a rate of 150 beats per minute. The patient was hemodynamically stable (blood pressure: 110/75). At the transthoracic exam, the K-wire appeared intra-pericardial, associated with a circumferential pericardial effusion, a collapse of the right atrium, and a dilated inferior vena cava. The chest computed tomography (CT) confirmed the intra-pericardial migration of one of the K-wires (Figs. 1 and 2). However, the measurement of the fluid density favored a reactive, non-hemorrhagic effusion.

The pin appeared laterally positioned toward the left of the mediastinum. Its most superficial point measured 50 mm from the skin surface.

Upon presentation of cardiac tamponade, urgent surgical intervention was warranted. Subsequently, a comprehensive multidisciplinary discussion involving the anesthesiologist, cardiothoracic surgeon, and cardiologist was undertaken to establish the optimal surgical approach. Given the patient's specific characteristics of obesity and diabetes, a meticulously planned minimally invasive strategy was chosen, involving a short anterolateral thoracotomy with a primary focus on addressing the wire while deliberately avoiding the need for a median sternotomy. However, it was collectively agreed that in the event of sudden hemodynamic deterioration, myocardial injury, or uncontrolled bleeding, an emergency sternotomy would be swiftly performed to address the critical situation. Therefore, a dedicated team of cardiac perfusionists, as well as a cardiopulmonary bypass machine, was available. The two Scarpas underwent surgical cleansing and were placed within the sterile field.

**Fig. 3:**
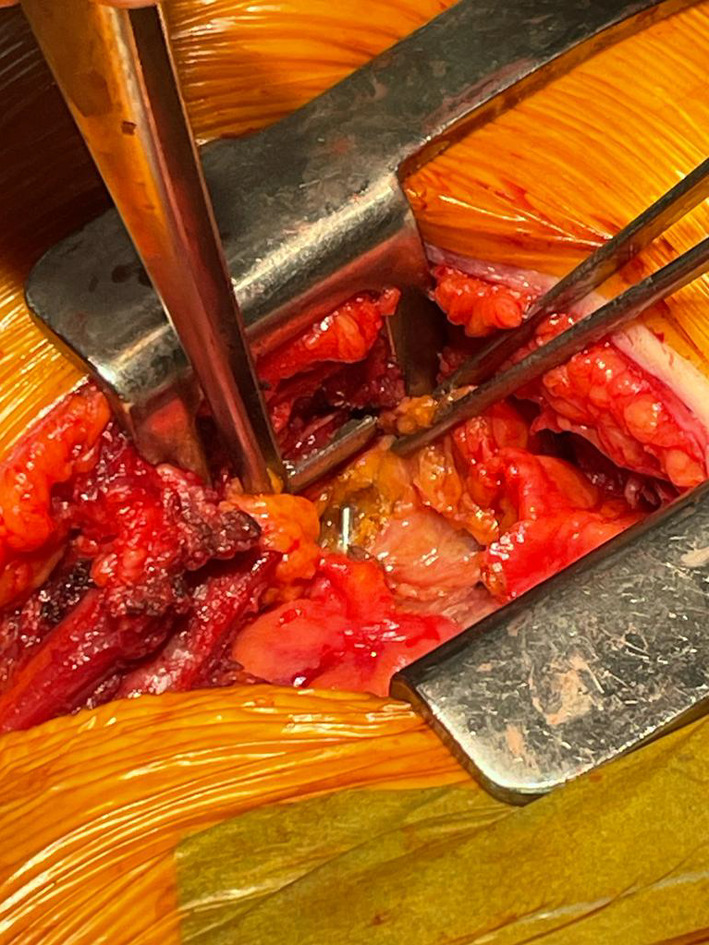
Per-operative view of the K-wire after dissection

Thus, the patient was positioned supine with the left arm at 90° and a bolster under the left flank. Due to the lack of reliable anatomical landmarks given the patient's body size, we utilized 2-plane orthogonal fluoroscopy (Siemens Cios Alpha device) to guide the skin incision (Additional file 1: Video S1). The initial step involved conducting an anteroposterior fluoroscopy to locate the position in a horizontal plane. This enabled us to make the incision centered on the pin, consisting in short left, 10 cm, anterior thoracotomy beneath the mammary fold. The initial step involved opening the left pleural cavity in the 6th intercostal space, resulting in the evacuation of 300 cc of reactive fluid. The dissection continued toward the anterior surface of the pericardium in the anterior mediastinum. With the assistance of fluoroscopy used in a lateral plane, the wire was quickly located. We proceeded with the opening of the pericardium, allowing for the evacuation of 250 cc of citrine fluid, and the wire was extracted (Additional file 2: Video S2, Fig. 3). No myocardial injury was found. The postoperative course was simple, allowing the removal of the pericardial and pleural drains on day 2. The patient was discharged on the 7th postoperative day.

## Discussion

This is the first case report describing the extraction of an intra-pericardial K-wire using a minimally invasive approach guided by fluoroscopy. The phenomenon of pin migration is well-known, and numerous case reports describe various migration sites, such as the inferior vena cava, lung, ascending aorta, and trachea [2]. There are multiple hypotheses proposed to explain these migrations. Factors such as muscular activity, breathing, and intra-thoracic negative pressure have been described. Insufficient fixation techniques have also been mentioned [3].

The site of K-wire implantation also determines the likelihood of migration. In a literature review, Freund found that most reported cases were associated with the sternoclavicular joint as the primary fixation location, followed by the clavicle, acromioclavicular joint, and proximal humerus [4]. Among approximately 50 reported cases in the literature, around 20 involved migration into the mediastinum, while 8 cases described an intra-cardiac. The exact migration trajectory remains uncertain. In our case, as in most articles found in the literature, the patient did not undergo any additional imaging assessments between the post-operative consultation and her urgent admission to our hospital.

In cases of intra-pericardial migration, the prognosis primarily depends on the presence of myocardial injury. In the case of heart or aortic injury, the outcome is invariably fatal [5].

Regardless, emergency sternotomy is considered the gold standard for diagnosis and emergent treatment. However, in our case, especially considering high-risk patients such as those with obesity and diabetes, our aim was to avoid this invasive approach in order to reduce postoperative complications (e.g., surgical site infection, osteitis, delayed wound healing).

The challenge was twofold: first, it was essential to ensure the absence of associated myocardial injury since the minimally invasive approach does not provide satisfactory control. Echocardiography, 3D reconstruction on thoracic CT scan, and measurement of pericardial fluid density assisted us in this regard.

Second, unlike the open and wide view provided by sternotomy, the approach through mini-thoracotomy limits the dissection. It was, therefore, crucial to locate the small-diameter pin perforating the pericardium within the adipose tissue of the anterior mediastinum. The use of continuous fluoroscopy in two orthogonal planes allowed us to guide the skin incision and progress the dissection centered on the pin. It is apparent that such technology is not universally available across all facilities. From our perspective, even single-plane fluoroscopy presents substantial advantages. While estimating pin depth might pose more challenges in anteroposterior fluoroscopy compared to biplanar methods, the procedure remains entirely viable.

## Conclusion

Kirschner wire migration is a known phenomenon. Although rare, migration in the intrathoracic position is almost systematically associated with severe lesions. In the event of migration, rapid surgical management is essential. The use of multimodal perioperative imaging such as CT scanner, TTE, and intra-operative X-ray, allows safe extraction via a minimally invasive approach.

### Supplementary Information

**Additional file 1: Video S1.** Continuous fluoroscopy centered on the thorax showing the K-wire.

**Additional file 2: Video S2.** Intra operative view of K-Wire removal.

## Data Availability

All medical data concerning the patient is available upon request.

## Abbreviations

K-wire: Kirschner wire
ECG: Electrocardiogram
CT: Computed tomography
TTE: Trans-thoracic echocardiogram
